# Yeast fungaemia among injection drug users in France (2012–2022): a cross-sectional observational study

**DOI:** 10.1016/j.lanepe.2025.101365

**Published:** 2025-06-25

**Authors:** Olivier Paccoud, Olivier Lortholary, Sebastien Imbert, Valerie Letscher-Bru, Karine Boukris-Sitbon, Thomas Obadia, Frederic Dalle, Lilia Hasseine, Florent Morio, Antoine Huguenin, Philippe Poirier, Loic Favennec, Julie Bonhomme, Alexandre Alanio, Fanny Lanternier, Marie Desnos-Ollivier, French Mycoses Study Group, Taieb Chouaki, Marc Pihet, Anne-Pauline Bellanger, Magalie Demar, Nicole Desbois-Nogard, Muriel Nicolas, Marie-Fleur Durieux, Milène Sasso, Estelle Perraud-Cateau, Jean-Pierre Gangneux, Caroline Mahinc, Sophie Cassaing, Adelaide Chesnay, Guillaume Desoubeaux, André Paugam, Elisabeth Chachaty, Marie-Elisabteth Bougnoux, Lilia Merabet, Patricia Mariani, Maité Micaelo

**Affiliations:** a Université Paris Cité, Department of Infectious Diseases and Tropical Medicine, Necker - Enfants Malades University Hospital, Assistance Publique – Hôpitaux de Paris (AP-HP), Paris, 75014, France; b Institut Pasteur, Université Paris Cité, National Reference Center for Invasive Mycoses and Antifungals, Mycology Translational Research Group, Mycology Department, Paris, France; c Service de Parasitologie - Mycologie, Centre Hospitalier Universitaire de Bordeaux, Bordeaux, F-33075, France; d Mycology-Parasitology Department, Hôpitaux Universitaires de Strasbourg, Strasbourg, France; e Institut de Parasitologie et de Pathologie Tropicale, UR3073 Pathogens-Host-Arthropods-Vectors Interactions, Université de Strasbourg, Strasbourg, France; f Mycology-Parasitology Department, Centre Hospitalier Universitaire (CHU) Dijon, Dijon, France; g Parasitologie - Mycologie, Hopital de l'Archet, CHU Nice, Nice, France; h Nantes Université, CHU Nantes, Cibles et Médicaments des Infections et de l'Immunité, Nantes, UR1155, France; i Université de Reims Champagne Ardenne, ESCAPE EA7510, Laboratoire de Parasitologie-Mycologie, Pole de Biologie Pathologie, CHU de Reims, Reims, France; j Université Clermont Auvergne, Inserm, 3IHP, Centre Hospitalier Universitaire Clermont-Ferrand, Service de Parasitologie-Mycologie, Clermont-Ferrand, France; k French National Cryptosporidiosis Reference Center, CHU de Rouen, Rouen, Normandie, France; l EA 7510, UFR Santé, University of Rouen Normandy, Rouen, France; m Laboratoire de Parasitologie-Mycologie, CHU de Caen, ToxEMAC-ABTE, Unicaen Université Normandie, Caen, France; n Laboratoire de Parasitologie-Mycologie, AP-HP, Hopital Saint-Louis, Paris, F-75010, France

**Keywords:** Yeast fungaemia, Candida, Injection drug use

## Abstract

**Background:**

Intravenous injection drug use (IVDU) is an established but infrequent risk factor for yeast fungaemia. We aimed to identify features and outcomes of yeast fungaemia associated with IVDU in a nationwide surveillance network in France.

**Methods:**

We prospectively included episodes of yeast fungaemia in adults between 2012 and 2022. Episodes with any history of IVDU were considered IVDU-associated. We compared clinical characteristics, infecting species, antifungal treatments, and 90-day mortality between groups. We used Fisher's exact test for categorical variables and the Kruskal–Wallis test for continuous variables.

**Findings:**

We recorded 9549 episodes of yeast fungaemia among 9132 adults. Among these, 183 (1·9%) were IVDU-associated. Compared with non-IVDU, individuals with IVDU-associated fungaemia were younger and less likely to have a history of malignancy (13·8%, 20/145 *vs.* 49·5%, 4447/8987, p < 0·0001) or recent surgery (24·3%, 34/140 *vs.* 37·3%, 3261/8,733, p = 0·0014), and more likely to have deep-seated infections (14·5%, 21/145 *vs.* 3·4%, 307/8987, p < 0·0001). IVDU cases less often involved *Candida albicans* (30·1%, 55/183 *vs.* 47·6%, 4458/9366, p < 0·0001) and more often involved mixed-species infections (13·1%, 24/183 *vs.* 4·3%, 402/9366), p < 0·0001. *Meyerozyma guilliermondii* and *Wickerhamomyces anomalus* were overrepresented among IVDU cases (6·0%, 11/183 *vs.* 0·5%, 48/9366, p < 0·0001 and 5·5%, 10/183 *vs.* 0·05%, 5/9366, p < 0·0001, respectively). The crude 90-day case-fatality ratio was 20·7% (95% CI: 13·7%–29·2%) in the IVDU group versus 48·4% (95% CI: 47·3–49·5%) for non-IVDU.

**Interpretation:**

IVDU-associated yeast fungaemia presents distinct clinical and microbiological characteristics, highlighting the need for tailored diagnosis and management.

**Funding:**

Financial support from Santé Publique France and 10.13039/501100003762Institut Pasteur, Paris.

**Research in context**

**Evidence before this study**

Yeast fungaemia is the most common invasive fungal infection globally. While haematological malignancy, solid-organ tumours, and recent surgery typically underlie most cases of yeast fungaemia, intravenous injection drug use (IVDU) has recently reemerged as a significant contributor in the United States amid the ongoing opioid epidemic. We searched PubMed for studies published from January 1st, 2004 to December 31st, 2024 using the terms “yeast”, “fung∗”, “candid∗”, “injection drug use”, and “intravenous drug use” without language restrictions. Previous single- or multistate population-based studies from the United States report a history of IVDU in up to 10% cases of candidaemia and have highlighted significant differences in patient characteristics according to IVDU status. To date, no such data have been reported for Europe, and specifically in France.

**Added value of this study**

We conducted a cross-sectional study nested within the French nationwide surveillance programme for invasive fungal infections (RESeau de Surveillance des Infections Fongiques invasives [RESSIF]), which includes 30 teaching hospitals across 12/13 current regions of France, and in 3/6 overseas departments and regions. We prospectively included all cases of yeast fungaemia from 2012 to 2022. Among 9549 cases of yeast fungaemia, 183 (1·9%) were associated with IVDU. Compared with non-IVDU patients, individuals with IVDU-associated fungemia were younger and less likely to have malignancies or a history of surgery, and more likely to be HIV-seropositive and exhibit end-organ involvement. IVDU-associated cases were more frequently associated with fungaemia recurrence, and more likely to be caused by non-albicans yeast species. Specifically, we identified a novel association between IVDU and fungaemia caused by *Wickerhamomyces anomalus* (syn. *Pichia anomala*), which was overrepresented among IVDU cases. Of note, 30-day and 90-day mortality rates were lower among IVDU-associated cases than in non-IVDU cases.

**Implications of all the available evidence**

Our study represents the largest cohort of patients with IVDU-associated yeast fungaemia and provides the first nationwide findings from Europe. IVDU-associated yeast fungaemia exhibits distinct clinical and microbiological characteristics compared with non-IVDU cases, which highlights the need for a tailored diagnostic and management approach that emphasises vigilance for end-organ complications, consideration of non-*albicans Candida* species, and strategies to prevent recurrence.

## Introduction

Yeast fungaemia is the most common invasive fungal infection globally and a leading cause of healthcare-associated bloodstream infection.^1^, ^2^, ^3^, ^4^, ^5^, ^6^ Typical risk factors for yeast fungaemia include haematological and solid-organ malignancies, abdominal surgery, and intensive care unit (ICU) stay.^7^^,^^8^ Intravenous injection drug use (IVDU) is a less common risk factor.^9^^,^^10^ In the 1980s and 1990s, outbreaks of candidaemia linked to IVDU were reported, with notably high rates of end-organ involvement.^9^^,^^11^ These outbreaks were linked to the injection of brown heroin, and specifically to the use of contaminated lemon juice to dissolve the drug.^12^ In addition to candidaemia, IVDU has also been associated with cases of disseminated cryptococcosis, as well as extra-pulmonary aspergillosis and mucormycosis.^10^^,^^13^, ^14^, ^15^, ^16^ Although the incidence and burden of candidaemia have remained significant over the following decades in France and globally,^7^^,^^17^ reports of IVDU-associated candidaemia cases have become more sporadic. However, recent reports from the United States have revealed concerning trends in IVDU-associated candidaemia amid the ongoing opioid epidemic, with upwards of 10% of cases of candidaemia in a multistate study in 2017 involving individuals with a history of IVDU.^18^ To date, no similar data on IVDU-associated fungaemia have been reported for Europe, and specifically in France.

In France, the National Centre for Invasive Mycoses and Antifungals (NRCMA, Institut Pasteur, Paris, France) conducts active nationwide surveillance for invasive fungal infections (RESeau de Surveillance des Infections Fongiques invasives [RESSIF]) and prospectively collects data on history of IVDU for all reported cases of yeast fungaemia. In this study, we aimed to determine the proportion of yeast fungaemia cases associated with IVDU in France from 2012 to 2022 and to compare the clinical features and species distribution between IVDU-associated and non-IVDU cases.

## Methods

We conducted a cross-sectional study nested within the RESSIF surveillance network, which comprises 30 teaching hospitals in 12/13 current regions of France, and in 3/6 overseas departments and regions (Supplementary Table S1). RESSIF is a prospective and nationwide active surveillance programme for all invasive fungal diseases, including yeast fungaemia, which was launched in January 2012. A detailed description of the surveillance programme was previously provided.^1^ Briefly, episodes of invasive fungal infections were declared by mycologists of the participating hospitals using an online questionnaire. This questionnaire included clinical and microbiological data (sex, date of birth, type of hospital ward, underlying conditions including IVDU, foreign devices, travel history, symptoms, imaging, diagnostic means, direct examination, culture, histology, PCR, specimens studied, prior antifungal prescription, initial therapy within the first 48 h, and 3-month outcome). Declarations were centralised and monitored at the NRCMA. The present study included all adult (≥18 years) patients with laboratory-confirmed yeast fungaemia between January 1st, 2012 and December 31st, 2022. A case was defined as a patient with a blood culture specimen yielding growth of any yeast species. At the NRCMA, cases of cryptococcosis, including those presenting with fungaemia, are captured in a separate surveillance system than other cases of yeasts, and were therefore not included. Both single and mixed-species episodes (>1 yeast species isolated from the same blood culture) were considered. Cases with any history of IVDU, regardless of recency, were classified as IVDU-associated cases and all others as non-IVDU cases. History of other types of injection, such as subcutaneous injection (skin-popping) is not routinely reported as part of surveillance and was therefore not collected. Recurrent episodes were defined as the isolation of the same species at least 10 days after an initial episode or of a different species at any time.^7^ Deep-seated infections were defined as cases of fungaemia in which the yeast was also isolated from a second anatomic site, except for respiratory or cutaneous specimen, which were considered indicative of colonisation. Community-onset cases were defined as those with a positive blood culture obtained ≤3 days after hospital admission.^19^

As part of this active national surveillance programme, yeast isolates were selectively referred to the NRCMA for species identification and susceptibility testing: only those that were either uncommon (*i.e.*, not among *Candida albicans*, *Nakaseomyces glabratus* [syn. *Candida glabrata*], *Candida tropicalis*, *Candida parapsilosis*, *Pichia kudriavzevii* [syn. *Candida krusei*], or *Kluyveromyces marxianus* [syn. *Candida kefyr*]) or displayed an unusual antifungal susceptibility profile were sent for review.^1^

The primary objective was to compare baseline characteristics and yeast species distribution according to IVDU status. Descriptive statistics were used to summarise demographic and clinical variables. To avoid autocorrelation, only incident cases were considered for comparisons of baseline characteristics between groups and case fatality ratios (CFR). Categorical variables were compared using Fisher's exact test, while continuous variables were compared using the Kruskal–Wallis test. Kaplan–Meier survival curves were plotted, and differences in 90-day mortality between IVDU and non-IVDU groups were compared using a log rank test.

Analyses were performed using Stata18 (StataCorp. Stata Statistical Software: Release 18. College Station, TX: StataCorp LLC).

### Reporting

STROBE guidelines were followed.^20^

### Ethics approval

NRCMA surveillance activities were approved by the Institut Pasteur Institutional Review Board 1 (2009–34/IRB) and the “Commission Nationale de l'Informatique et des Libertés” (2004-903395).

### Role of the funding source

This research was supported by Institut Pasteur, Paris and Santé Publique France. The funders had no role in study design, data collection and analysis, decision to publish, or preparation of the manuscript.

## Results

Between January 1st, 2012 and December 31st, 2022, a total of 9549 cases of yeast fungaemia were recorded among 9132 adult individuals within the RESSIF nationwide surveillance network. We categorised 183 (1·9%) episodes as IVDU-associated, while 9366 (98·1%) occurred in non-IVDU individuals. Of these, 38/183 (20·8%) from the IVDU group and 379/9366 (4·0%) from the non-IVDU group were recurrent episodes (p < 0·0001). No discernible temporal or geographical trends in case distribution were observed, with the annual proportion of IVDU-associated cases ranging from 1·5% in 2022 to 3·1% in 2014 (Supplementary Figure S1).

Patient characteristics according to IVDU status for the 9132 individuals with incident fungaemia are summarised in Table 1. Patients with IVDU-associated fungaemia were younger (median age: 45 years, 25th, and 75th centiles [Q1–Q3]: 38–51 years) than patients with non-IVDU fungaemia (66 years [Q1–Q3: 56–75 years], p < 0·0001). Patients in the IVDU group were less likely to have typical risk factors for yeast fungaemia, such as solid-organ or haematological malignancies (13·8%, 20/145 *vs.* 49·5%, 4447/8987, p < 0·0001) or a history of recent surgery (24·3%, 34/140 *vs.* 37·3%, 3261/8733, p = 0·0014), and more likely to be HIV-seropositive (12·3%, 13/106 *vs.* 3·6%, 121/3354, p = 0·00013). Notably, a history of IVDU was identified in 3·4% (87/2536) of individuals otherwise without an identified risk factor for fungaemia, and in 13·0% (46/354) of those 19–44 years and without another risk factor. The median time from hospital admission to positive blood culture was significantly shorter in IVDU-associated cases than non-IVDU cases (5 days [Q1–Q3: 0–18 days] *vs.* 11 days [Q1–Q3: 3–23 days], p < 0·0001) and IVDU-associated cases were more likely to be community-acquired (46·5%, 67/144, *vs.* 28·2%, 2478/8793, p < 0·0001). Among those with available data, patients with IVDU-associated fungaemia were more likely to have a prosthetic cardiac valve (20·8%, 11/53 *vs.* 4·7%, 196/4137, p < 0·0001) and to have deep-seated infections (14·5%, 21/145 *vs.* 3·4%, 307/8987, p < 0·0001). Of note, 28·0% (14/50) of all individuals recorded as having endocarditis during this period had a history of IVDU. Finally, IVDU cases were more likely to be treated with antifungal combination therapy (9·2%, 13/141 *vs.* 2·4%, 213/8726, p = 0·00028).

**Table 1:** Baseline characteristics, clinical features, and antifungal treatment according to history of intravenous injection drug use (IVDU) among 9132 adults with incident yeast fungaemia (2012–2022).

Variables	All participants (n = 9132)	IVDU (n = 145)	Non-IVDU (n = 8987)	p-value
Age				
Median age (Q1–Q3)	66 (55–75)	45 (38–51)	66 (56–75)	<0·0001
19–45 years	1057/9132 (11·6%)	71/145 (49·0%)	986/8987 (11·0%)	<0·0001
45–64 years	3177/9132 (34·8%)	68/145 (46·9%)	3109/8987 (34·6%)	
≥65 years	4898/9132 (53·6%)	6/145 (4·1%)	4892/8987 (54·4%)	
Male sex	5711/9132 (62·5%)	116/145 (80·0%)	5595/8987 (62·3%)	<0·0001
Recent surgery	3295/8873 (37·1%)	34/140 (24·3%)	3261/8733 (37·3%)	0·0014
Abdominal surgery	1611/3289 (49·0%)	10/34 (29·4%)	1601/3255 (49·2%)	0·025
Indwelling vascular catheter	6302/77,673 (82·1%)	78/102 (76·5%)	6224/77,571 (82·2%)	0·15
Prosthetic cardiac valve	207/4190 (4·9%)	11/53 (20·8%)	196/4137 (4·7%)	<0·0001
History of haematological malignancy	1458/8977 (16·2%)	7/142 (4·9%)	1451/8835 (16·4%)	<0·0001
History of solid-organ malignancy	3122/8978 (34·8%)	14/142 (9·9%)	3108/8836 (35·2%)	<0·0001
HIV seropositive	134/3581 (3·7%)	13/106 (12·3%)	121/3354 (3·5%)	0·00013
Deep-seated infections	328/9132 (3·6%)	21/145 (14·5%)	307/8987 (3·4%)	<0·0001
Culture-confirmed endocarditis	50/9132 (0·5%)	14/145 (9·7%)	36/8987 (0·4%)	<0·0001
Culture-confirmed endophthalmitis	74/9132 (0·8%)	2/145 (1·4%)	72/8987 (0·8%)	0·33
Culture-confirmed osteoarticular involvement	33/9132 (0·4%)	3/145 (2·1%)	30/8987 (0·3%)	0·015
In ICU at diagnosis	3353/9040 (37·1%)	50/143 (35·0%)	3303/8897 (37·1%)	0·66
Antifungal preexposure				0·049
None	8439/9124 (92·5%)	141/145 (97·2%)	8298/8979 (92·4%)	
Polyene	44/9124 (0·5%)	1/145 (0·7%)	43/8979 (0·5%)	
Echinocandin	258/9124 (2·8%)	0/145 (0%)	258/8979 (2·9%)	
Azole	383/9124 (4·2%)	3/145 (2·1%)	380/8979 (4·2%)	
Median [Q1–Q3] time from admission to positive blood culture (days)^a^	11 (3–23)	5 (0–18)	11 (3–23)	<0·0001
Community acquired	2519/8937 (28·2%)	67/144 (46·5%)	2478/8793 (27·9%)	<0·0001
First-line antifungal treatment				0·00028
No antifungals^b^	1118/8867 (12·6%)	11/141 (7·8%)	1107/8726 (12·7%)	
Polyene monotherapy	195/8867 (2·2%)	1/141 (0·7%)	194/8726 (2·2%)	
Echinocandin monotherapy	5295/8867 (59·7%)	88/141 (62·4%)	5207/8726 (59·7%)	
Azole monotherapy	2033/8867 (22·9%)	28/141 (19·9%)	2005/8726 (23·0%)	
Combination therapy	226/8867 (2·6%)	13/141 (9·2%)	213/8726 (2·4%)	
30-day case fatality ratio	3038/8328 (36·5%)	18/125 (14·4%)	3020/8203 (36·8%)	<0·0001
90-day case fatality ratio	3796/7909 (48·0%)	24/116 (20·7%)	3772/7793 (48·4%)	<0·0001

Notes. HIV, human immunodeficiency virus; ICU, intensive care unit; IVDU, intravenous injection drug use; Q1–Q3, 25th and 75th centiles.

a Available data: n = 8937.

b Due to post-mortem diagnosis in 3/11 (27·3%) of IVDU cases and 489/1107 (44·2%) of non-IDU cases.

The distribution of infecting species is detailed in Table 2. *C. albicans* was the predominant species, accounting for 47·3% (4513/9549) of cases overall. However, among incident and recurrent cases, those associated with IVDU were significantly less likely to be caused by *C. albicans* (30·1%, 55/183 *vs.* 47·6%, 4458/9366, p < 0·0001) and more often involved mixed-yeast infections (13·1%, 24/183 *vs.* 4·3%, 405/9366, p < 0·0001). Of note, infections caused by *Meyerozyma guilliermondii* and *W. anomalus* were particularly overrepresented in IVDU cases compared to non-IVDU cases (6·0%, 11/183 *vs.* 0·5%, 48/9366, p < 0·0001 and 5·5%, 10/183 *vs.* 0·05%, 5/9366, p < 0·0001, respectively). Among mixed-species infections, *W. anomalus* was isolated in 2/20 (10%) incident and 2/4 (50%) recurrent IVDU-associated cases, always co-occurring with *M. guilliermondii*.

**Table 2:** Details of yeast species associated with 9549 cases of incident or recurrent yeast fungaemia.

Yeast species	Incident episodes			Recurrent episodes		
	IVDU-associated cases (n = 145)	non-IVDU cases (n = 8987)	p-value^a^	IVDU-associated cases^b^ (n = 38)	non-IVDU cases^c^ (n = 379)	p-value
*Candida albicans*	51/145 (35·2%)	4332/8987 (48·2%)	0·0018	4/38 (10·5%)	126/379 (33·3%)	0·0030
*Candida parapsilosis*	19/145 (13·1%)	1055/8987 (11·7%)	0·60	7/38 (18·4%)	80/379 (21·1%)	0·84
*Nakaseomyces glabratus*	19/145 (13·1%)	1548/8987 (17·2%)	0·22	2/38 (5·3%)	70/379 (18·5%)	0·042
*Meyerozyma guilliermondii*	7/145 (4·8%)	46/8987 (0·5%)	<0·0001	4/38 (10·5%)	2/379 (0·5%)	0·00078
*Wickerhamomyces anomalus*	7/145 (4·8%)	4/8987 (0·04%)	<0·0001	3/38 (7·9%)	1/379 (0·3%)	0·0026
*Candida tropicalis*	6/145 (4·1%)	595/8987 (6·6%)	0·31	6/38 (15·8%)	22/379 (5·8%)	0·032
*Clavispora lusitaniae*	3/145 (2·1%)	152/8987 (1·7%)		1/38 (2·6%)	12/379 (3·2%)	
*Candida dubliniensis*	3/145 (2·1%)	81/8987 (0·9%)		–	2/379 (0·5%)	
*Rhodotorula mucilaginosa*	3/145 (2·1%)	45/8987 (0·5%)		1/38 (2·6%)	4/379 (1·1%)	
*Kluyveromyces marxianus*	2/145 (1·4%)	142/8987 (1·6%)		1/38 (2·6%)	3/379 (0·8%)	
*Pichia kudriavzevii*	1/145 (0·7%)	252/8987 (2·8%)		3/38 (7·9%)	–	
*Candida fermentati*	1/145 (0·7%)	8/8987 (0·1%)		–	1/379 (0·3%)	
*Candida metapsilosis*	1/145 (0·7%)	26/8987 (0·3%)		–	2/379 (0·5%)	
*Debaryomyces hansenii*	1/145 (0·7%)	1/8987 (0·01%)		–	–	
*Debaryomyces fabryi*	1/145 (0·7%)	–		–	–	
*Candida quercitrusa*	–	–		2/38 (5·3%)	–	
Other species	–	322/8987 (3·6%)		–	27/379 (7·1%)	
Mixed species	20/145 (13·8%)	378/8987 (4·2%)	<0·0001	4/38 (10·5%)	27/379 (7·1%)	0·51
*C. albicans*	9/20 (45·0%)	228/378 (60·3%)		2/4 (50·0%)	12/27 (44·4%)	
*C. dubliniensis*	7/20 (35·0%)	17/378 (5·5%)		–	2/27 (7·4%)	
*C. glabrata*	6/20 (30·0%)	163/378 (43·1%)		1/4 (25·0%)	8/27 (29·6%)	
*C. tropicalis*	6/20 (30·0%)	62/378 (16·4%)		1/4 (25·0%)	6/27 (22·2%)	
*C. parapsilosis*	3/20 (15·0%)	68/378 (18·0%)		–	6/27 (22·2%)	
*M. guilliermondii*	3/20 (15·0%)	8/378 (2·1%)		2/4 (50·0%)	–	
*Clavispora lusitaniae*	2/20 (10·0%)	19/378 (5·0%)		–	1/27 (3·7%)	
*P. kudriavzevii*	2/20 (10·0%)	47/378 (12·4%)		–	5/27 (18·5%)	
*W. anomalus*	2/20 (10·0%)	1/378 (0·3%)		2/4 (50·0%)	–	
Other mixed	–	10/378 (2·6%)		–	4/27 (14·8%)	

Note. IVDU, intravenous injection drug use.

a Comparisons between groups were only performed for the 6 most frequently identified species among IVDU cases and for mixed-species infections.

b 38 cases in 19 individuals (median 4, [Q1–Q3, 2–7], range: 1–8 recurrent cases per individual).

c 379 cases in 336 individuals (median 2 [Q1–Q3, 2–2], range: 1–8 recurrent cases per individual).

**Fig. 1:**
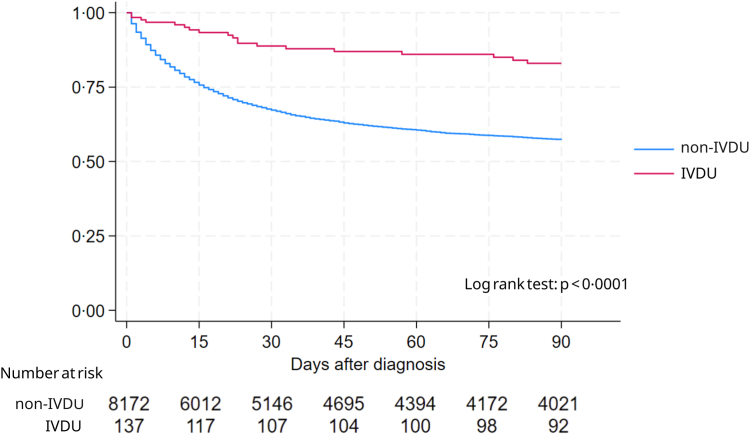
Kaplan–Meier survival estimates up to day 90 according to intravenous injection drug use (IVDU) status. Note. IVDU, intravenous injection drug use.

Of 7907 individuals with incident fungaemia and available outcome data, the crude 90-day CFR was 20·7% (95% CI: 13·7%–29·2%) among individuals in the IVDU group and 48·4% (95% CI: 47·3–49·5%) among individuals in the non-IVDU group. A difference in mortality was observed after 2 days, and survival curves did not overlap up to day 90 (log rank test p-value of <0·0001) (Fig. 1). Similarly the crude 30-day CFR was 14·4% (95% CI: 8·8%–21·8%) among individuals in the IVDU group and 36·8% (95% CI: 35·8–37·9%) among individuals in the non-IVDU group.

## Discussion

In this nationwide surveillance study in France, we found that IVDU was associated with 1·1–2·8% of yeast fungaemia cases per year over an eleven-year period. This is substantially lower than the 10–12% previously reported in single- or multistate studies from the United States.^18^^,^^21^, ^22^, ^23^ In the United States, which has seen a dramatic increase in rates of opioid use and overdose deaths over the past two decades,^24^ there is evidence that IVDU is reemerging as a significant risk factor for candidaemia amid the ongoing opioid crisis.^18^^,^^21^, ^22^, ^23^ Recent estimates from 2021 are of 14·8 million people injecting drugs globally, with marked variations by region, sex, and age groups.^25^ According to the 2023 European Drug Report, France ranked 7th in Europe for IVDU prevalence (2·7 per 1000 population) over the 2015–2021 period and had the highest absolute number of individuals who injected drugs.^26^ However, unlike trends in the United States,^18^^,^^22^ we did not observe an increase in IVDU-associated fungaemia over an eleven-year surveillance period. This aligns with evidence from drug treatment centres indicating a decline in injection as the primary route of drug administration in Europe among new users over the past decade.^26^

Previous studies from the United States have highlighted differences in characteristics of patients with fungaemia according to IVDU status.^18^^,^^21^^,^^22^ Individuals with IVDU-associated fungaemia are younger than those with non-IVDU fungaemia, are predominantly male, and have fewer traditional risk factors such as malignancy or recent surgery. In addition, we found that patients with IVDU are more likely to be HIV-seropositive, to have community-onset fungaemia, and to have end-organ complications such as endocarditis. The latter is consistent with previous data from France on *Candida* sp. endocarditis, where up to 30% of affected patients were injection drug users.^27^

As previously reported,^18^^,^^22^ we found that mortality rates among IVDU were markedly lower than for non-IVDU. This should be attributed to the younger age of IVDU and the absence of comorbidities usually associated with yeast fungaemia, rather than to a lower infection severity in these individuals.^10^ Indeed, it is important to note that IVDU-associated fungaemia is associated with significant morbidity, particularly related to secondary complications such as endocarditis. In addition, yeast fungaemia in non-IVDU is generally associated with severely compromised immunity and underlying conditions with high attributable mortality.^7^^,^^8^ This could also reflect differences in pathophysiology. Specifically, IVDU were presumably more likely to be hospitalised for the fungaemia event, while a significant proportion of non-IVDU may have already been acutely ill due to the underlying condition at the time of fungaemia. Finally, IVDU-associated fungaemia likely results from a transient inoculation event rather than from a persistent source of infection, which may also help explain differences in yeast species distribution.

Using a comprehensive surveillance programme with species-level identification, we also identified significant differences in the distribution of infecting species between IVDU and non-IVDU cases. While *C. albicans* was the main species isolated, we found a higher prevalence of non-*albicans* yeast species among IVDU cases compared to non-IVDU cases. Although not statistically significant, IVDU cases had lower proportions of *N. glabratus* and *C. tropicalis*, species more commonly associated with older patients and malignancy.^8^ Conversely, we also found a relatively high proportion of cases caused by yeast species known to colonise skin, such as *C. parapsilosis* and *M. guilliermondii*. This particular association between *M. guilliermondii* and IVDU was previously reported in multistate data from the United States, where *M. guilliermondii* accounted for 3·6% of IVDU-associated cases of candidemia compared to only 0·7% of non-IVDU cases.^18^ Here, we also report a novel association between IVDU and *W. anomalus*, which accounted for 5·5% of incident or recurrent cases among IVDU compared to only 0·05% of non-IVDU cases. Determining whether this association reflects a specific source of infection or a common at-risk behaviour requires further studies, ideally incorporating environmental sampling. However, unlike *C. albicans* outbreaks linked to contaminated brown heroin in the 1980s–1990s,^9^^,^^11^ the absence of temporal trends, as well as the large diversity in infecting species found in this study may argue against a common source of infection for these cases of IVDU-associated yeast fungemia. Regardless, given the high proportion of recurrent cases of yeast fungemia in the IVDU group compared to non-IVDU group, this report highlights the importance of harm-reduction interventions in this population (for e.g., syringe exchange programmes, opioid agonist treatment, or supervised consumption facilities).^28^

Some limitations are due to the data collected in the French surveillance network. As the participating centres are not representative of the French regions, and as the number of centres has changed over the study period, we could not accurately evaluate changes in the incidence of IVDU-associated fungaemia over time. It is likely that the number of IVDU-associated cases may be underestimated in the surveillance database. Indeed, the study was not designed to specifically capture IVDU and patients may not have disclosed all IVDU history, or it may have been missed by the clinicians. In addition, we could not collect data on whether the injection drug use was current or not, so it is possible that some patients with past drug use and receiving opioid substitution treatments were misclassified in the IVDU group. However, as these opioid substitution treatments can also be misused, we chose to include all patients with a history of IVDU in the IVDU group, even if remote. Similarly, we could not collect data on the nature of drugs injected, nor on specific at-risk behaviours associated with IVDU. Finally, we could not assess the impact of harm-reduction measures and factors associated with fungemia recurrence among IVDU cases.

**Fig. 2:**
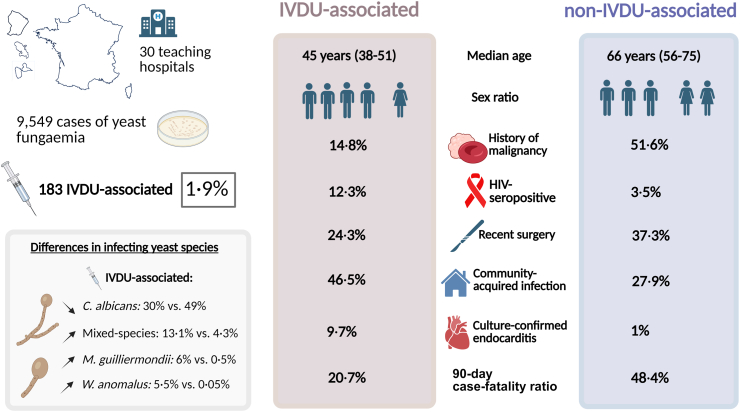
Graphical abstract. Note. HIV, human immunodeficiency virus; IVDU, intravenous injection drug use.

In conclusion, IVDU was associated with only a small proportion of cases of yeast fungemia in a large surveillance programme in France. However, patient characteristics, distribution of species involved, and mortality in these IVDU-associated cases differ significantly from those of non-IVDU cases (Fig. 2), highlighting the need for a tailored diagnostic and management approach that emphasises vigilance for end-organ complications, consideration of non-*albicans Candida* species, and strategies to prevent recurrence. As patterns of drug use in Europe may be changing and with projected increases in synthetic opioid use,^29^ ongoing surveillance programmes for IVDU-associated fungaemia remain of the utmost importance.

## Contributors

Writing – Original Draft: O.P.; Writing – Review & Editing: S.I., T.O., A.A., O.L., F.L., M.D.O.; Methodology: O.P., T.O., F.L., M.D.O.; Data acquisition & curation: K.B.S., F.D., V.L.B., L.H., F.M., A.H., P.P., L.F., J.B., A.A., French Mycoses Study Group; Conceptualisation: O.P., O.L., S.I., F.L., M.D.O.; Access to data: O.P., F.L., M.D.O.; Decision to publish: O.P., F.L., M.D.O.

## Data sharing statement

We support data sharing of the deidentified participant data in accordance with FAIR principles. Request for access should be directed beginning 3 months and ending 1 year after publication to Fanny Lanternier (fanny.lanternier@pasteur.fr). These proposals will be reviewed and approved on the basis of scientific merit and in accordance with the principles of Institut Pasteur Ethics Charter (https://www.pasteur.fr/sites/default/files/rubrique_linstitut_pasteur/nos_engagements/ethique/ethics_charter_pasteur-2022.pdf). The request concerns the deidentified participant data that underlie the results reported in this article, after de-identification (text, tables, figures, and appendix), and software code. Data will be available at the CNRMA in Pasteur Institute, Paris, France. To obtain access, data requesters will need to sign a data access agreement and use the secure data sharing tools provided by Institut Pasteur, Paris.

## Editor note

The Lancet Group takes a neutral position with respect to territorial claims in published maps and institutional affiliations.

## Declaration of interests

A.A. reports an issued patent in a subject outside this work. All other authors have nothing do declare.

## Appendix A Supplementary data

Supplementary Table and Figure

French Mycoses Study Group

Biorender_Publication License May-31-2025
