# Supplementary material for: Yeast fungaemia among injection drug users in France (2012–2022): a cross-sectional observational study
Source: Lancet Reg Health Eur. 2025 Jun 25;55:101365. doi: 10.1016/j.lanepe.2025.101365 (PMC12426822; doi:10.1016/j.lanepe.2025.101365)
Supplement: Supplementary Table and Figure [file mmc1.docx]

**Supplementary Materials**

**Table of contents**

**Figure S1 -** Total number of intravenous injection drug use (IVDU) cases (a), and proportion of IVDU cases among total cases reported (b), per year.  **p.2**

**Table S1 –** List of centres, cities, and French regions in the RESSIF surveillance network. **p.3**

**Figure S1.** Total number of intravenous injection drug use (IVDU) cases (a), and proportion of IVDU cases among total cases reported (b), per year.

**
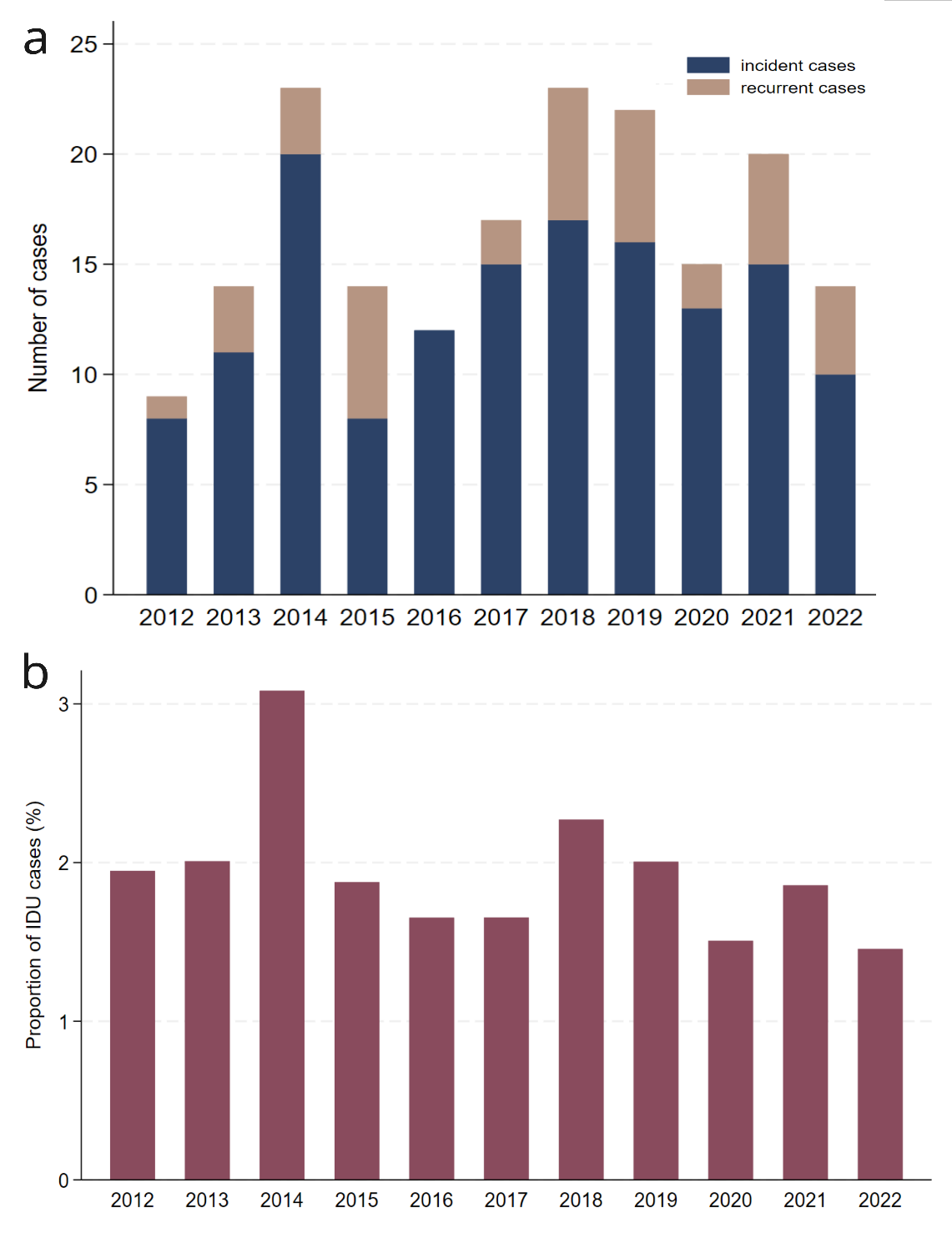
**

**Table S1. List of centres, cities, and French regions in the RESSIF surveillance network.**

| **Hospital** | **City** | **French Administrative Region** |
| --- | --- | --- |
| CHU Clermont Ferrand | Clermont-Ferrand | Auvergne Rhone Alpes |
| CHU St Etienne | Saint Etienne | Auvergne Rhone Alpes |
| CHU Besançon | Besançon | Bourgogne Franche Comté |
| CHU Dijon | Dijon | Bourgogne Franche Comté |
| CHU Rennes | Rennes | Bretagne |
| CHU Orléans | Orléans | Centre Val de Loire |
| CHU Tours | Tours | Centre Val de Loire |
| CHU Reims | Reims | Grand Est |
| CHU Strasbourg | Strasbourg | Grand Est |
| CHU Guadeloupe | Pointe à Pitre | Guadeloupe |
| CHU Guyane | Cayenne | Guyane |
| CHU Amiens-Picardie | Amiens | Haut de France |
| GHU Saint Louis-Lariboisière, AP-HP | Paris | Ile de France |
| Hopital André Mignot-CH Versailles | Le Chesnay | Ile de France |
| Hopital Cochin-Port Royal, AP-HP | Paris | Ile de France |
| Hopital National des 15-20 | Paris | Ile de France |
| Hopital Necker Enfants Malades, AP-HP | Paris | Ile de France |
| Hopital Robert Debré, AP-HP | Paris | Ile de France |
| Institut Gustave Roussy | Villejuif | Ile de France |
| CHU Martinique | Fort de France | Martinique |
| CHU Caen-Normandie | Caen | Normandie |
| CHU Rouen-Normandie | Rouen | Normandie |
| CHU Limoges | Limoges | Nouvelle Aquitaine |
| CHU Poitiers | Poitiers | Nouvelle Aquitaine |
| CHU Montpellier | Montpellier | Occitanie |
| CHU Nimes | Nimes | Occitanie |
| CHU Toulouse | Toulouse | Occitanie |
| CHU Angers | Angers | Pays de la Loire |
| CHU Nantes | Nantes | Pays de la Loire |
| CHU Nice | Nice | Provence Alpes Cote d'Azur |

Note. CHU, Centre hospitalo-universitaire; GHU, groupe hospitalo-universitaire
