# Supplementary material for: Yeast fungaemia among injection drug users in France (2012–2022): a cross-sectional observational study
Source: Lancet Reg Health Eur. 2025 Jun 25;55:101365. doi: 10.1016/j.lanepe.2025.101365 (PMC12426822; doi:10.1016/j.lanepe.2025.101365)
Supplement: French Mycoses Study Group [file mmc2.docx]

| **First name** | **Surname** | **Affiliation** |
| --- | --- | --- |
| Taieb | Chouaki | Laboratoire de Parasitologie et Mycologie Médicales, Centre de Biologie Humaine, CHU Amiens Picardie, Amiens, France. |
| Marc | Pihet | Laboratoire de Parasitologie-Mycologie, Hôpital Universitaire Angers, Angers, France. |
| Anne-Pauline | Bellanger | Laboratoire de Parasitologie-Mycologie, Centre hospitalier universtaire (CHU) Besançon, Besançon, France |
| Magalie | Demar | Laboratoire Hospitalo-Universitaire de Parasito-Mycologie, Centre hospitalier de Cayenne Guyane, Cayenne, France |
| Nicole | Desbois-Nogard | Laboratoire de Parasitologie-Mycologie, Centre Hospitalier Universitaire de Martinique, Fort-de-France, Martinique, France. |
| Muriel | Nicolas | Laboratoire de Parasitologie-Mycologie, Centre Hospitalier Universitaire de Guadeloupe, Pointe-à-Pitre, Guadeloupe, France. |
| Marie-Fleur | Durieux | Laboratoire de Parasitologie-Mycologie, CHU Dupuytren, Limoges, France |
| Milène | Sasso | Laboratoire de Parasitologie-Mycologie, CHU Nîmes & Université de Montpellier, CNRS, IRD, MiVEGEC, Montpellier, France. |
| Estelle | Perraud-Cateau | Laboratoire de Parasitologie-Mycologie, Hôpital Universitaire Poitiers, INSERM U1070, Université de Poitiers, France. |
| Jean-Pierre | Gangneux | Université de Rennes, CHU Rennes, Inserm, EHESP, Irset (Institut de recherche en santé, environnement et travail) - UMR_S 1085, Laboratory of Parasitology and Medical Mycology, European Confederation of Medical Mycology (ECMM) Excellence Center, Centre, Rennes, France National de Référence Aspergilloses Chroniques, Rennes Teaching Hospital, F-35000 Rennes, France |
| Caroline | Mahinc | Mycology-Parasitology Department, CHU Saint Etienne, Saint-Priest-En-Jarez, France |
| Sophie | Cassaing | Department of Parasitology and Mycology, Toulouse University Hospital, Restore-FLAMES, Toulouse III University, France |
| Adelaide | Chesnay | Centre Hospitalo-Universitaire Régional de Tours, Department of Parasitology-Mycology-Tropical Medicine, Tours, France. |
| Guillaume | Desoubeaux | Centre Hospitalo-Universitaire Régional de Tours, Department of Parasitology-Mycology-Tropical Medicine, Tours, France. |
| André | Paugam | Laboratoire de Parasitologie-Mycologie, Centre Hospitalier Universitaire Cochin, Assistance Publique-Hôpitaux de Paris, Paris, France |
| Elisabeth | Chachaty | Medical Biology Department, Institut Gustave Roussy, Villejuif, France |
| Marie-Elisabteth | Bougnoux | Parasitology-Mycology Unit, Necker Enfants Malades Hospital, APHP, Paris, France |
| Lilia | Merabet | Laboratoire de Biologie, Hopital National des Quinze-Vingts, Paris, France |
| Patricia | Mariani | CHU Robert Debré, Paris, France |
| Maité | Micaelo | Mycology-Parasitology Department, Hôpital André Mignot, le Chesnay-Rocquencourt. |
